# Neuroprotective Potential of Intranasally Delivered Sulforaphane-Loaded Iron Oxide Nanoparticles Against Cisplatin-Induced Neurotoxicity

**DOI:** 10.1007/s12640-022-00555-x

**Published:** 2022-08-15

**Authors:** Ghadha Ibrahim Fouad, Sara A. M. El-Sayed, Mostafa Mabrouk, Kawkab A. Ahmed, Hanan H. Beherei

**Affiliations:** 1 Department of Therapeutic Chemistry, Pharmaceutical and Drug Industries Research Institute, National Research Centre, 33 El-Bohouth St., Dokki, Cairo, 12622 Egypt; 2 Refractories, Ceramics and Building Materials Department, Advanced Materials Technology and Mineral Resources Research Institute, National Research Centre, 33 El Bohouth St., Dokki, Cairo, 12622, Egypt; 3 Pathology Department, Faculty of Veterinary Medicine, Cairo University, 12211 Giza, Egypt

**Keywords:** Cisplatin, Sulforaphane, Iron oxide nanoparticles, Intranasal, Neurotoxicity, Acetylcholinesterase

## Abstract

Cisplatin (CIS) is a platinum-based chemotherapeutic drug that is widely used to treat cancer. However, its therapeutic efficiency is limited due to its potential to provoke neurotoxicity. Sulforaphane (SF) is a natural phytochemical that demonstrated several protective activities. Iron oxide nanoparticles (Fe_3_O_4_-NPs) could be used as drug carriers. This study aimed to explore the nanotoxic influence of SF-loaded within Fe_3_O_4_-NPs (N.SF), and to compare the neuroprotective potential of both N.SF and SF against CIS-induced neurotoxicity. N.SF or SF was administrated intranasally for 5 days before and 3 days after a single dose of CIS (12 mg/kg/week, i.p.) on the 6^th^ day. Neuromuscular coordination was assessed using hanging wire and tail-flick tests. Acetylcholinesterase (AChE) activities and markers of oxidative stress were measured in the brain. In addition, the brain iron (Fe) content was estimated. CIS significantly induced a significant increase in AChE activities and lipid peroxides, and a significant decrement in glutathione (GSH) and nitric oxide (NO) contents. CIS elicited impaired neuromuscular function and thermal hyperalgesia. CIS-induced brains displayed a significant reduction in Fe content. Histopathological examination of different brain regions supported the biochemical and behavioral results. Contradict, treatment of CIS-rats with either N.SF or SF significantly decreased AChE activity, mitigated oxidative stress, and ameliorated the behavioral outcome. The histopathological features supported our results. Collectively, N.SF demonstrated superior neuroprotective activities on the behavioral, biochemical, and histopathological (striatum and cerebral cortex) aspects. N.SF could be regarded as a promising “pre-clinical” neuroprotective agent. Furthermore, this study confirmed the safe toxicological profile of Fe_3_O_4_-NPs.

## Introduction

Cisplatin (CIS) is a widely used chemotherapeutic agent for the treatment of different malignancies including testicular, head, neck, ovarian, cervical, and several other types of cancers (Chiorazzi et al. 2015). CIS exerts its therapeutic action through provoking oxidative stress and apoptosis in tumor cells (Pabla and Dong 2008). Despite its therapeutic efficiency in reducing tumor burden, CIS was found to be extremely cytotoxic to off-target tissues; CIS could enhance nephrotoxicity, neurotoxicity, ototoxicity, and cardiotoxicity (Shabani et al. 2012a, b; Hussein et al. 2020).

Cisplatin is the most neurotoxic agent among the platinum-based compounds (Shabani et al. 2012a, b; Kazak et al. 2021); CIS-induced neurotoxicity is dose-dependent (Frisina et al. 2016). CIS can suppress neurogenesis and damage nerve cells and oligodendrocytes (Manohar et al. 2014). Despite its poor ability to cross the blood–brain barrier (BBB), CIS is capable of stimulating morphological alterations in the white matter structure and disrupting neurotransmitters (Zhou et al. 2016; Owoeye et al. 2018). CIS-induced neurotoxicity might trigger neuroinflammation due to the ability of the pro-inflammatory cytokines, to cross the BBB, induce neuroinflammatory responses, and disrupt the integrity of the BBB (Wardill et al. 2016; Ren et al. 2017).

Additionally, CIS-associated peripheral neuropathy (PN) is one of the most relevant and dose-limiting side effects (Argyriou et al. 2012); CIS is capable of stimulating demyelination in both large and small myelinated fibers (Boehmerle et al. 2014). CIS-induced PN is clinically manifested as numbness, pain, decreased vibratory sensitivity, and declined ankle jerk reflex (Akbar et al. 2020). CIS-receiving cancer patients might suffer from incomplete recovery and require a prolonged duration to restore neural function (Carozzi et al. 2015); this might be ascribed to the CIS-associated “coasting” phenomenon, where the symptoms might last for prolonged months and can progressively worsen over time, even after the cessation of CIS administration (Siegal and Haim 1990), thus affecting the quality of life of cancer patients.

The presence of BBB prevents the entrance of potential therapeutic drugs into the brain (Veronesi et al. 2020). Therefore, the respiratory or olfactory biodistribution pathways of drugs from the nasal mucosa to the neural tissues are currently considered alternative and non-invasive delivery methods that facilitate the passage of drugs through the BBB (Pires and Santos 2018). The intranasal (IN) route of administration (nose to brain drug delivery) enables the direct delivery of bioactive therapeutic molecules to the CNS (Singh et al. 2016). Furthermore, IN administration is patient-friendly, simple, non-expensive, and safe, and could be achieved by introducing the drugs in the cleft of the nasal cavity and subsequent absorption in the nasal mucosa (Dhuria et al. 2010). In addition, the olfactory route is more efficient for decreasing hepatic and renal clearance and the systemic exposure (Khan et al. 2017). Models of nasal drug delivery can be used for toxicological studies by testing nasal drug absorption and permeation (Erdő et al. 2018).

One of the emerging strategies in terms of improvement of the bioavailability and efficacy of nutraceuticals is the development of nanodelivery (Panzarini et al. 2020). The use of nanocarriers as a novel drug delivery tool has gained much attention in the management of neural disorders due to their “site-specific targeting” potential (Md et al. 2018). Pure iron oxides such as magnetite (Fe_3_O_4_) and maghemite (γ-Fe_2_O_3_) are the most commonly used biocompatible magnetic nanomaterials (Ling and Hyeon 2013). Iron oxides (IO) are benign, nontoxic, and tolerated biologically; furthermore, IO could be incorporated into humane natural metabolism when used for drug delivery systems (Luo et al. 2020). Magnetic iron oxide nanoparticles (Fe_3_O_4_-NPs) have recently attracted a lot of attention due to their unique magnetic and physicochemical features, and their promising nanotheranostic application in the biomedical fields, including brain imaging and brain-targeted delivery (Mabrouk et al. 2021). The biomedical application of iron oxide nanoparticles (IONPs) as nanotechnology-based cancer therapeutics and/or diagnostics will pave the way for the advancement of cancer nanomedicine (Rosen et al. 2012; Dadfar et al. 2019).

Magnetite NPs contribute to developing drug delivery because of their unique properties including their bioavailability and high surface-to-volume ratio, improving the contact between the medication and the cerebrospinal fluid (CSF) (Busquets et al. 2015). Neurons are especially vulnerable to cytotoxicity; thus, there is a developing interest in creating magnetic nanoparticle (MNP) details and magnetofection protocols (Lamkowsky et al. 2012). The potential cytotoxic influences of free iron ions released from IONPs can be inhibited by maintaining iron homeostasis (Luo et al. 2020). The toxicity profile of IONPs could be estimated by their physicochemical features (e.g., size, shape, concentration, surface charge) (Yarjanli et al. 2017). Furthermore, the inappropriate features of most of the drugs or bioactive molecules including poor solubility and nonspecific delivery could be surpassed by the use of IONPs for drug delivery (Parveen et al. 2012).

Sulforaphane (SF) is a natural powerful polyphenol that characterizes seeds and sprouts of cruciferous plants such as broccoli; SF is a natural “isothiocyanate” widely studied for its pleiotropic potential on several disease models (Calcabrini et al. 2020); SF exhibited antioxidative and protective potential in different disorders such as focal cerebral ischemia, ischemia–reperfusion, induced acute renal failure, and neurotoxicity (Gaona-Gaona et al. 2011; Guerrero-Beltrán et al. 2012). SF showed neuroprotective activities against traumatic brain and spinal cord injury (Benedict et al. 2012; Klomparens and Ding 2019; Nadeem et al. 2019). This protective potential of SF could be ascribed to its function as “an indirect reactive oxygen species (ROS) scavenger”; SF is capable of upregulating phase II biotransformation enzymes by stimulating nuclear factor E2-related factor 2 (Nrf2). In the nucleus, Nrf2 functions as a transcription activator for antioxidant response elements (ARE), (Briones-Herrera et al. 2018).

The current study aimed to investigate the toxicological profile of intranasally (IN) administrated SF-loaded within Fe_3_O_4_-NPs (N.SF), as well as, to compare the neurotherapeutic potential of loaded and molecular “free” forms of SF against CIS-induced central and peripheral neurotoxicity in rats through behavioral, biochemical, and histopathological investigations of different brain regions.

## Materials and Methods

### Drugs

Cisplatin^®^ vial (50 mg/25 ml injectable solution) was purchased from Mylan S.A.S. (Saint-Priest, France). dl-Sulforaphane (SF) (5 mg, ≥ 90% (HPLC), synthetic, liquid (CAS number: 4478–93-7), was purchased from Sigma (St. Louis, USA). The anhydrous iron chloride (FeCl_3_) (97%) (MWt = 162.20 g/mol) was purchased from Sigma (St. Louis, MO, USA). SF and SF-loaded Fe_3_O_4_-NPs (N.SF), dispersed in phosphate buffer saline (PBS), were stored at 4 °C during the experimental use.

### Chemicals

The anhydrous iron chloride (FeCl_3_) (97%) (MWt = 162.20 g/mol) was purchased from Sigma-Aldrich (Germany). Ethylene glycol ((CH_2_OH)_2_) (99%) (MWt = 62.07 g/mol) was purchased from Alpha (India). Hydrated hydrazine (N_2_H_4_·H_2_O) (99%) (MWt = 50.06 g/mol) was purchased from Advent (India). Absolute ethanol (C_2_H_5_O) (99.8%) (MWt = 46.07 g/ mol) was purchased from Piochem (Egypt). Tween 80 was purchased from Sigma (St. Louis, MO, USA). Acetylcholinesterase (AChE) activity was estimated using commercially available ELISA kits according to the manufacturer’s instructions (Biovision, USA), while colorimetric kits of lipid peroxides (LPO), glutathione reduced (GSH), and nitric oxide (NO) were purchased from Biodiagnostic Co*.*, Egypt.

### Preparation of Magnetic Iron Oxide Nanoparticles (Fe_3_O_4_-NPs)

Magnetic iron oxide (IO) was prepared as mentioned before in our research paper with few modifications (Mabrouk et al. 2021). In detail, a yellowish-brown suspension mixture of FeCl_3_ with ethylene glycol (60 ml) was prepared by continuous agitation at room temperature. This was followed by the addition of hydrated hydrazine (6 ml) and kept under the above-mentioned conditions until the solution color turned dark brown. Continuing stirring for 1 h, the solution color was returned to yellowish-brown again. At that time, the solution was transferred into a Teflon vessel of stainless steel autoclave and kept at 150 °C for 48 h. Then, the autoclave was cooled down to room temperature and the resultant solution was filtrated and washed with both distilled and ethanol using 6000 rpm centrifugation velocity for 20 min, and the black precipitates were collected and dried for 48 h at 60 °C afterward.

### Sulforaphane (SF) Loading Within Magnetic Iron Oxide Nanoparticles (Fe_3_O_4_-NPs)

SF (5 mg) was dissolved in a small volume (250 μl) of Tween 80 (Sigma, St. Louis, MO, USA) and diluted to the appropriate concentration with phosphate buffer saline (PBS) (10.4 ml); the final concentration of Tween 80 did not exceed 1%. In this mixture, 100 mg of Fe_3_O_4_-NPs was dispersed using an ultrasonic homogenizer. The resulted mixture was vortexed before IN administration to rats in order to ensure the homogenous N.SF distribution.

### TEM Analysis

Transmission electron microscopy (TEM) (JEOL, Japan, JEM-2100, electron microscope, TEM-HR) was used to identify the particle size of the Fe_3_O_4_-NPs before and after SF loading. Suspensions of Fe_3_O_4_-NPs free and SF-loaded using ethanol were prepared, then 5 μl of each suspension was dropped on a copper grid and, images were further recorded by TEM after the grids were dried in air.

### Particle Size Distribution and Zeta Potential

The size distribution and the zeta potential of the prepared Fe_3_O_4_-NPs, before and after SF loading, were determined using the method of light scattering using the Zetasizer Nano ZS instrument (Malvern Instruments, UK, accessorized with a 633 nm laser). Samples were dispersed in a clear disposable zeta cell by using deionized water at 25 °C. The measuring position in the case of measuring the size distribution is 5.50 mm from the wall of the zeta cuvette and 2.00 mm in the case of measuring the zeta potential. Malvern instrument’s dispersion technology software (version 7.13) was used for data analysis and zeta potential.

### In Vivo Study

#### Experimental Animals

**Fig. 1:**
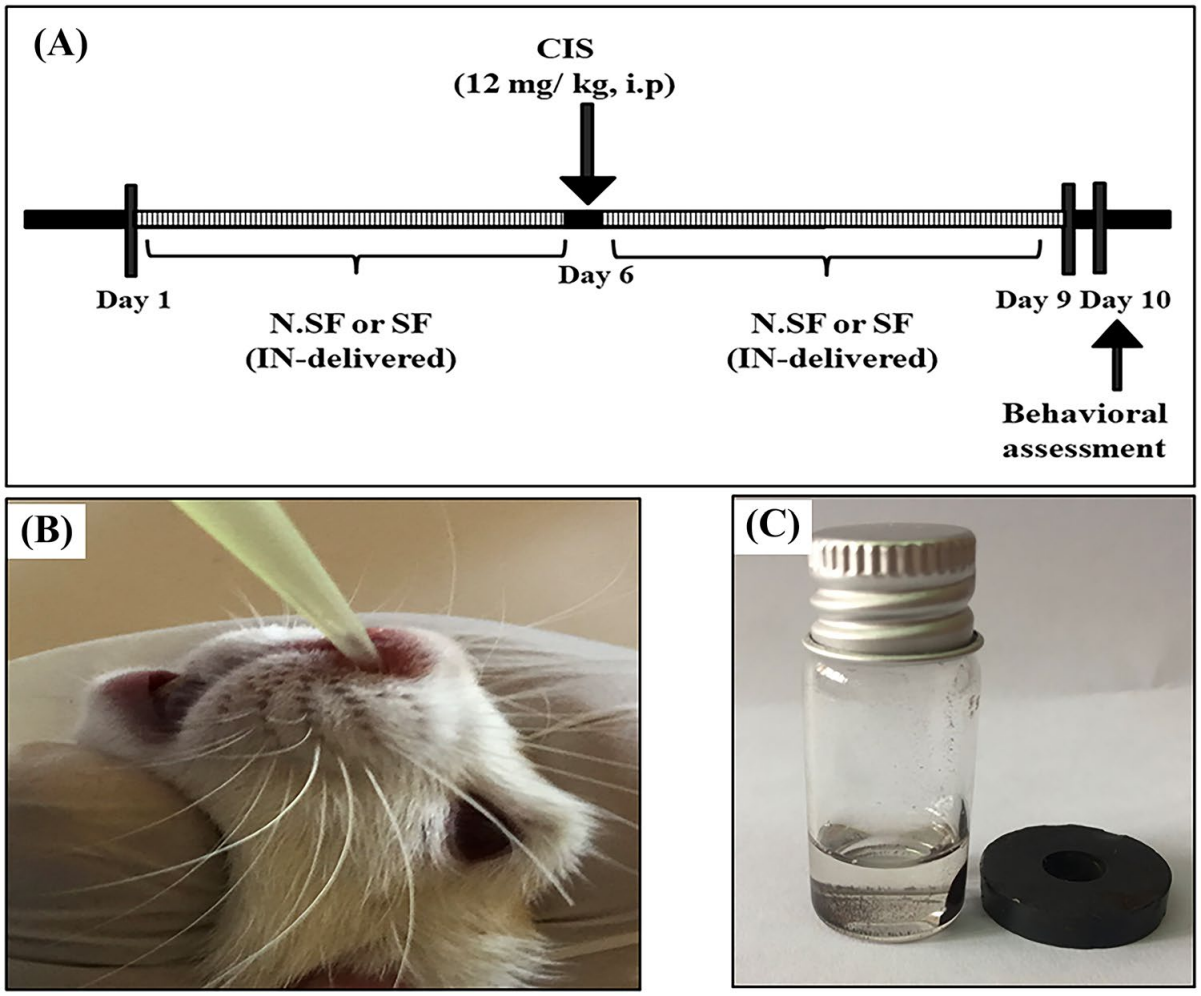
**A** Schematic representation of timeline and experimental design, **B** intranasal dosage (IN) administration in rats, and **C** demonstration of magnetic nature of iron oxide nanoparticles (Fe_3_O_4_-NPs) used for SF loading

Adult female albino Wistar rats were used in the present work of age 6–8 weeks and mean weight of 130 ± 10 g. The animals were kept in standard cages in groups of five animals in each cage under fixed housing conditions (12-h light/dark cycles) and temperature (25 ± 1 °C). The water and chow were provided ad libitum. This study was performed in line with the principles of the Declaration of Helsinki. Approval was granted by the Ethics Committee of the National Research Centre (NRC), Egypt (approval no. 19–313). The in vivo experimental design is presented in Fig. 1A.

#### Intranasal (IN) Administration and Selected Dose

IN administration was performed using a polyethylene tip attached to a micropipette, and inserted approximately 5 mm into the right nostril without anesthesia (Fig. 1B). Both SF and N.SF were dissolved or suspended in PBS (pH 7.4) (Fig. 1C). Rats were held in a supine position, and then 25 μl of either N.SF or SF formulations was instilled gently and slowly into the nasal cavity using a micropipette every other day. The nostril was observed closely for signs of blockage or irritation. The dose of 2 mg/kg of SF was determined according to Saleh et al. (2019).

#### Experimental Design

Twenty-five rats were divided randomly into five groups (*n* = 5):

**Group (1)**: Negative control rats: intranasally administrated 25 μl PBS into the right nostril for 9 days, interrupted by a single intraperitoneal (i.p.) dose of distilled water on the 6^th^ day.

**Group (2)**: CIS-neurotoxicated positive control rats: received a single i.p. dose of CIS (12 mg/kg), on the 6^th^ day of the experiment (Khadrawy et al. 2019).

**Group (3)**: N.SF-treated alone control rats: N.SF was intranasally administrated for 9 days; each rat received 25 μl Fe_3_O_4_-NPs containing 2 mg/kg SF.

**Group (4)**: CIS + N.SF-treated rats: N.SF was intranasally administrated for 5 days followed by a single i.p. dose of CIS (12 mg/kg) on the 6^th^ day, followed by 3 successive days of IN administration with N.SF.

**Group (5)**: CIS + SF-treated rats: free SF was intranasally administrated for 5 days followed by a single i.p. dose of CIS (12 mg/kg) on the 6^th^ day, followed by 3 successive days of IN administration with SF.

The first set of behavioral tests was performed 24 h after the end of the experimental period.

### Behavioral Assessment

#### Evaluation of Locomotor and Neuromuscular Coordination: Hanging Wire (Grip) Test

The hanging wire test was conducted using a simple manual apparatus according to Van Wijk et al. (2008) to evaluate neuromuscular balance. Each rat, held in a vertical position, was lifted by the tail and suspended with both forepaws on a horizontal steel wire (25 cm long, diameter 10 mm). When the rat grasped the wire and hung using its forelimbs, the operator gently pulled its tail backward. Then, the rat was released, and the latency to fall was recorded with a stopwatch. Rats were randomly tested and each rat was given three trials with a 30-min inter-trial rest interval.

#### Assessment of Thermal Hyperalgesia: Tail-Flick Test

To analyze the spinal thermal sensitivity, the withdrawal threshold after thermal (hot and cold water) stimulation of the rat tail using the “tail immersion test” was measured. The withdrawal latency was defined as "the time (seconds) between immersion of tail in hot or cold water (with a cut-off time of 15 s) and the time of withdrawal" (Necker and Hellon 1977). Tail thermal hyperalgesia was noted with the dipping of the terminal (1 cm) of the tail in water maintained at a temperature of 50 ± 1.0 °C or ice-cold water, until the manifestation of signs of pain including tail flicking, shaking, withdrawal, or struggle. The time elapsed for the first display of pain or the tail-flick reflex was recorded.

### Brain Tissue Sampling and Preparation

The rats were sacrificed by cervical dislocation under sodium thiopental anesthesia (100 mg/kg). The whole-brain tissue was dissected and washed with normal saline. The right half of each brain area was weighed and homogenized in 5 ml ice-cold PBS (50 mM, pH 7.4) using a glass-Teflon homogenizer, centrifuged at 6000 rpm for 20 min using a high-speed cooling centrifuge, and the clear supernatant obtained after centrifugation was frozen at −20 °C to be utilized for the estimation of biochemical parameters of AChE, LPO (MDA), GSH, and NO. The left half of each brain sample was fixed at 10% buffered formalin for 24 h and specified for histopathological investigation.

### Biochemical Analyses

#### Determination of Brain Acetylcholinesterase (AChE) Activities

Brain AChE activity was estimated by ELISA as per the manufacturer’s manual. The absorption of the yellow-colored product was read at 412 nm. Results were expressed as pg/100 mg brain tissue.

#### Determination of Brain Contents of Lipid Peroxides (LPO)

Brain LPO or malondialdehyde (MDA), a measure of lipid peroxidation, was assayed by measuring the thiobarbituric acid reactive substances according to Ohkawa et al. (1979). The thiobarbituric acid reactive substances (TBARS) interact with thiobarbituric acid to produce a pink-colored complex having absorbance at 534 nm which was detected by a UV/Vis spectrophotometer. The results were expressed as nmol/g of wet tissue.

#### Determination of Brain Contents of Reduced Glutathione (GSH)

Brain GSH contents were determined according to Beutler et al. (1963). The method is based on the reduction of 5,5-dithiobis-2-nitrobenzoic acid (DTNB) by “-SH” groups of GSH to form the yellow-colored “2-nitro-s-mercaptobenzoic acid” having absorbance at 405 nm which was detected by a UV/Vis spectrophotometer. The results were expressed as mg/g of wet tissue.

#### Determination of Brain Contents of Nitric Oxide (NO)

Brain NO contents were estimated according to Montgomery and Dymock (1961). Nitrate was reduced to nitrite using the nitrate reductase enzyme. This was followed by an assay of the nitrite using Griess reagent that converts nitrite into a purple azo-compound, which was measured spectrophotometrically at 540 nm against a blank. NO concentration was expressed as µmol/g of wet tissue.

#### Iron (Fe) and Platinum (Pt) Quantification in Brain Tissues

Prior to metal determination, brain tissues were digested according to the analytical method of APHA (2017). Brain contents of iron (Fe) and platinum (Pt) were quantified in brain tissues of different groups. After excision and dissection, brain samples were digested using a 1:3 volumetric mix of hydrochloric acid to nitric acid. Digestions were incubated at 70 °C for up to 12 h until all tissues had gone into solution and a clear liquid is obtained. Determination of Fe and Pt contents was quantified using an inductively coupled plasma optical emission spectrometer (ICP-OES) (Agilent 5100 Synchronous Vertical Dual View (SVDV) ICP-OES, with Agilent Vapor Generation Accessory VGA 77, Agilent Technologies). For each series of measurements, intensity calibration curve was constructed composed of a blank and three or more standards from Merck Company (Germany). Accuracy and precision of the metal measurements were confirmed using external reference standards from Merck, and standard reference material for trace elements in water and quality control sample from the National Institute of Standards and Technology (NIST) were used to confirm the instrument reading. The detection limit of the instrument was 0.01 ppb.

### Histopathological Investigation

Brain specimens were collected from different groups and fixed in 10% neutral buffered formalin. Paraffin sections of 5-μm thickness were prepared and stained with hematoxylin and eosin (H&E) (Suvarna et al. 2019) for histopathological examination and inspected blindly by the pathologist under a light microscope (BX43, Olympus) and photographed using the Cellsens dimension software (Olympus) connected to a Olympus DP27 camera. Neuropathological damage was graded from 0 to 4 as follows: (0) indicated no changes; (1) indicated percentage area affected (< 10%); (2) indicated percentage area affected (20–30%); (3) indicated percentage area affected (40–60%); and (4) indicated percentage area affected (> 60%) (Farag et al. 2021).

### Statistical Analysis

Results were expressed as mean ± SEM (standard error of the mean) and were analyzed using the Duncan test as a post hoc test with a significance level (*p* < 0.05). Statistical Package for the Social Sciences (SPSS, version 25) origin software was used for all data. The percent of the difference in the value of data for the treated group with respect to the negative or positive CIS control was also calculated and presented as a percentage difference. Percentage of change % = (treated value − control value) / control value × 100.

## Results

### TEM Analysis of N.SF (SF-Loaded Fe_3_O_4_-NPs)

**Fig. 2:**
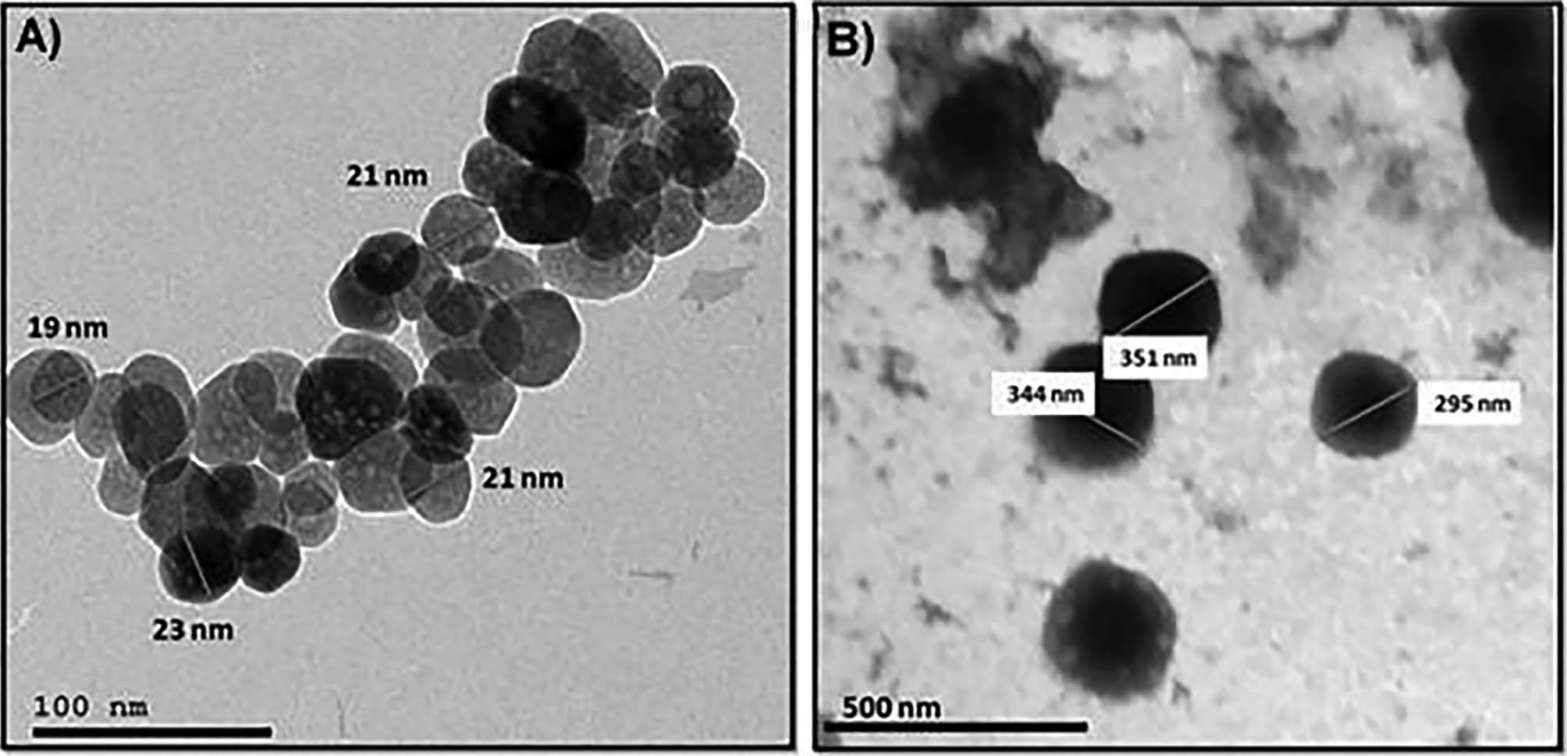
TEM images of (**A**) magnetic iron oxide nanoparticles (Fe_3_O_4_-NPs) and (**B**) sulforaphane (SF)-loaded iron oxide nanoparticles (Fe_3_O_4_-NPs)

TEM analysis was performed to confirm the morphology of the prepared Fe_3_O_4_-NPs before and after SF loading. TEM image (Fig. 2A) showed that the prepared Fe_3_O_4_-NPs have a nanosphere particle–like shape that demonstrated a diameter range between 19 and 23 nm. Figure 2B demonstrates the SF-loaded Fe_3_O_4_-NPs and illustrates the change of their size to 295–344 nm. Different illumination degrees observed for the SF-loaded samples indicate the formation of core–shell formulation type, in which the lighter area (the outer boundaries) is related to SF-loaded molecules and the darker zone (core part) is attributed to the Fe_3_O_4_-NPs.

### Particle Size Distribution and Zeta Potential of N.SF (SF-Loaded Fe_3_O_4_-NPs)

**Fig. 3:**
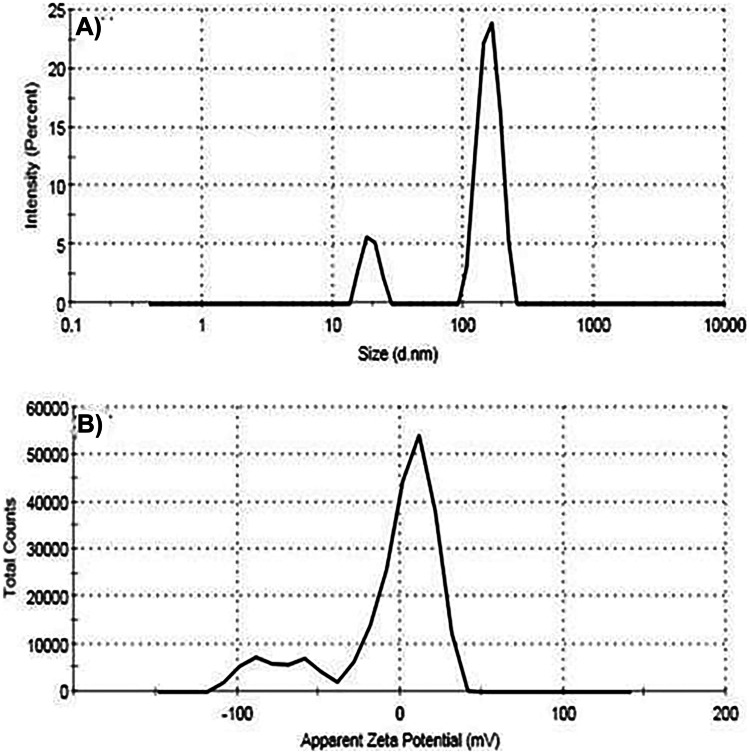
**A** Particle size distribution curve of the prepared sulforaphane (SF) loaded within iron oxide nanoparticles (Fe_3_O_4_-NPs) and **B** zeta potential profile of the prepared sulforaphane (SF) loaded within iron oxide nanoparticles (Fe_3_O_4_-NPs) recorded by the Zetasizer device

Size distribution and zeta potential were carried out using the Zetasizer instrument to confirm the cell interaction with nanoparticles that were affected by its charge. Figure 3A shows the particle size distribution of SF-loaded Fe_3_O_4_-NPs, which has exhibited an average dynamic distribution of particle diameters of 339 nm with 100% intensity. Zeta potential (Fig. 3B) of SF-loaded Fe_3_O_4_-NPs has a maximum zeta potential value at − 7.18 mV.

**Fig. 4:**
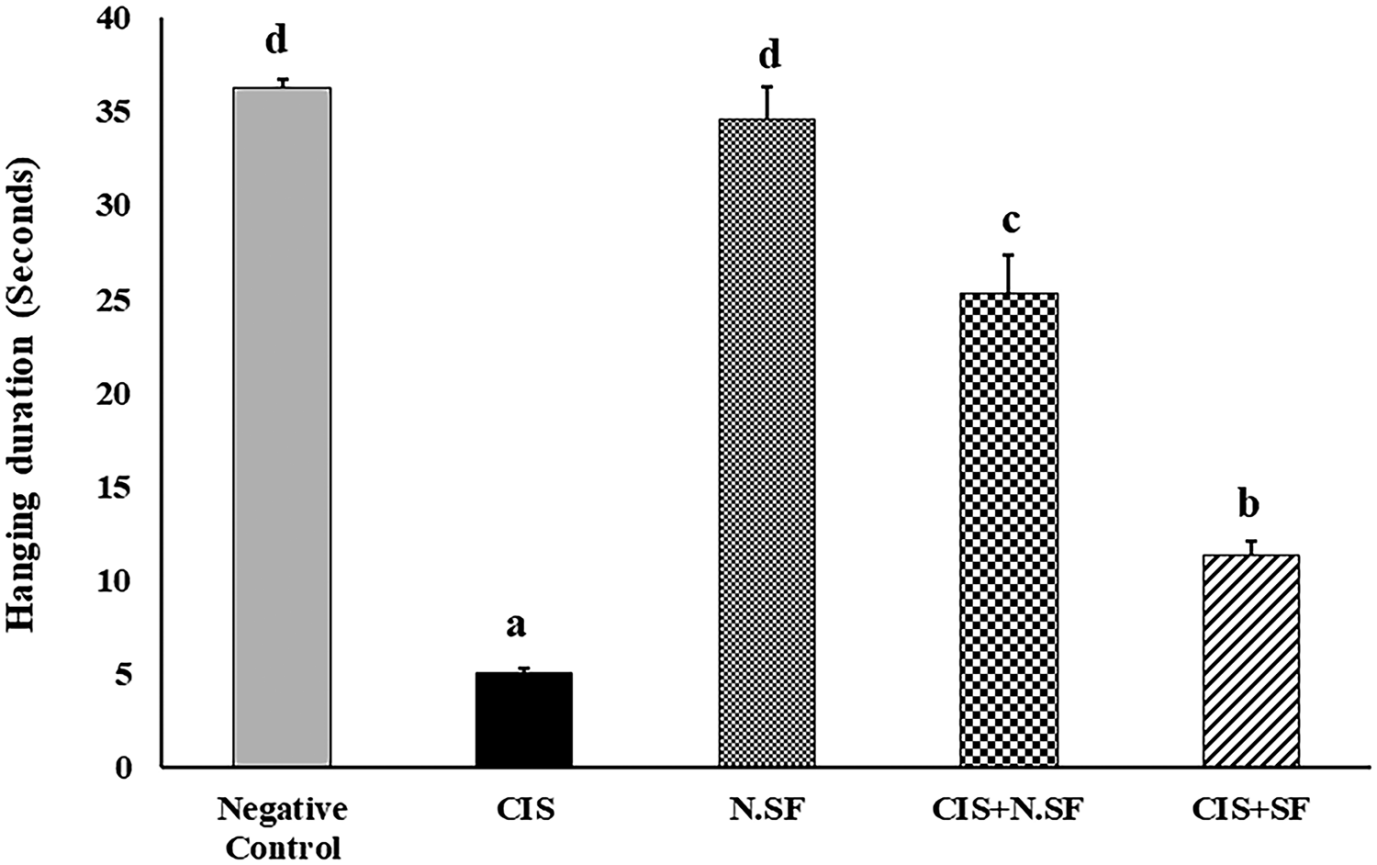
Effect of intranasal administration of either sulforaphane (SF)-loaded within iron oxide nanoparticles (N.SF) or free sulforaphane (SF) on neuromuscular coordination “hanging wire” test in CIS-induced rats. Groups: negative control rats, cisplatin (CIS)-exposed rats, N.SF-alone exposed rats, CIS + N.SF-treated rats, and CIS + SF-treated rats. Results are presented as mean ± standard error of the mean (SEM). Mean with different letters (a–d) is significant at *p* ≤ 0.05

**Fig. 5:**
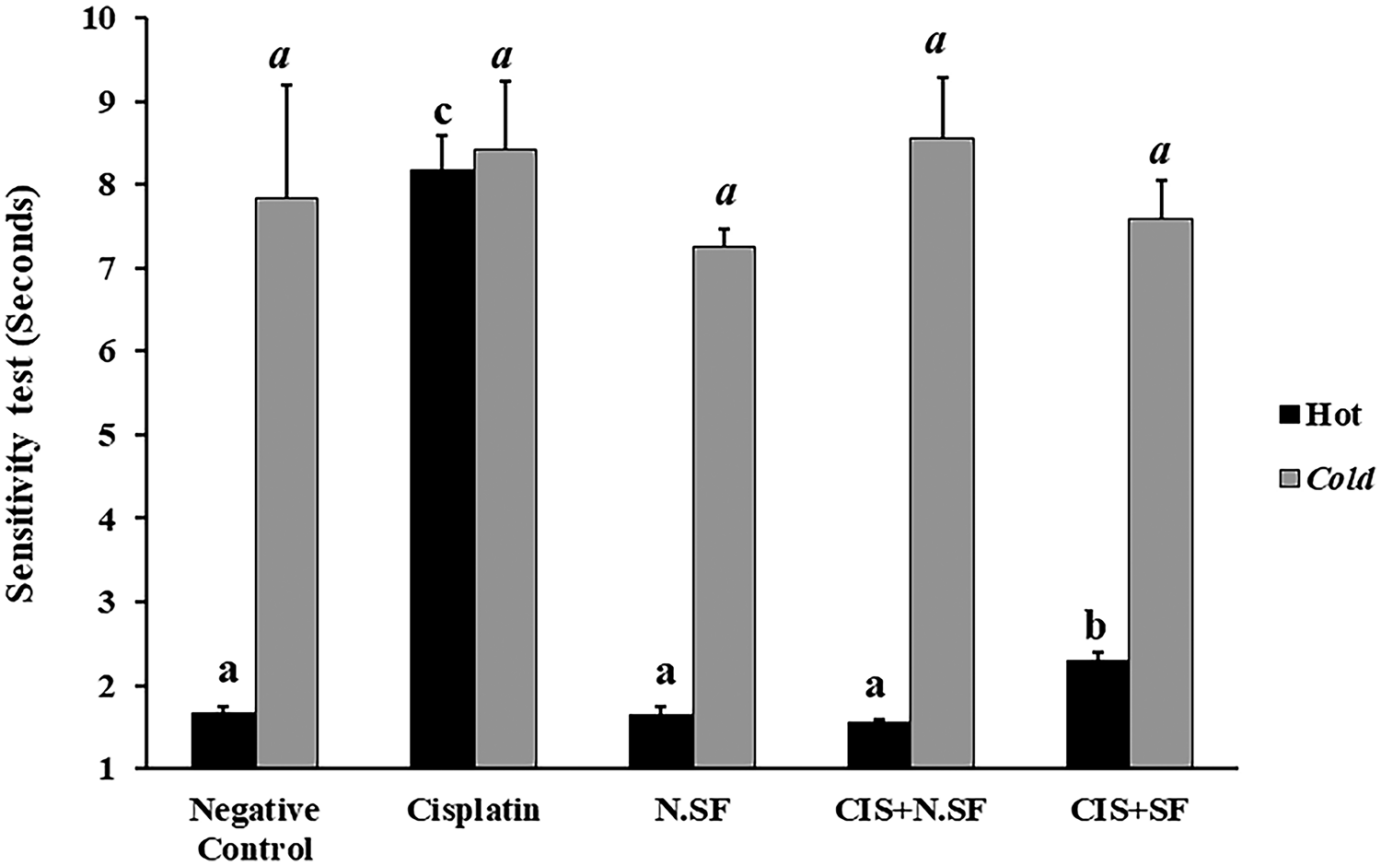
Effect of intranasal administration of sulforaphane (SF)-loaded within iron oxide nanoparticles (N.SF) or free sulforaphane (SF) on thermal sensitivity: tail-flick test in CIS-induced rats. Groups: negative control rats, cisplatin (CIS)-exposed rats, N.SF-alone exposed rats, CIS + N.SF-treated rats, and CIS + SF-treated rats. Results are presented as mean ± standard error of the mean (SEM). Mean with different letters (a–c) is significant at *p* ≤ 0.05

**Fig. 6:**
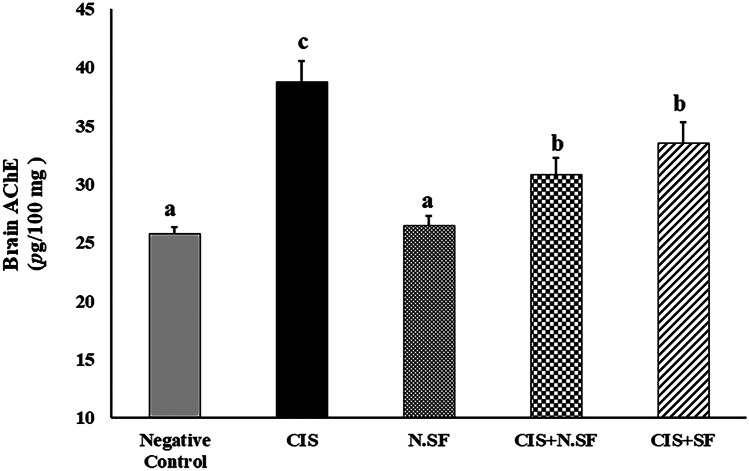
Effect of intranasal administration of sulforaphane (SF)-loaded within iron oxide nanoparticles (N.SF) or free sulforaphane (SF) on brain acetylcholinesterase (AChE) activities in CIS-induced rats. Groups: negative control rats, cisplatin (CIS)-exposed rats, N.SF-alone exposed rats, CIS + N.SF treated rats, and CIS + SF-treated rats. Results are presented as mean ± standard error of the mean (SEM). Mean with different letters (a–c) is significant at *p* ≤ 0.05

### Effect of IN-Delivered N.SF or SF on Neuromuscular Coordination in CIS-rats

The hanging wire test was performed to assess the neuromuscular coordination and balance in rats of different experimental groups. CIS-induced rats demonstrated a significant decrease in time elapsed for wire hanging by 86.15%, indicating reduced latency to fall, as compared to negative rats. On the other side, IN administration of either N.SF or SF to CIS-rats increased the time elapsed for wire hanging by 405.78% and 126.2%, respectively, as compared to CIS-rats, signifying increased latency to fall. N.SF-alone rats showed an insignificant change to negative control rats. These findings showed that IN-administrated N.SF improved neuromuscular coordination and balancing better than SF in CIS-induced rats, as illustrated in Fig. (4).

### Effect of IN-Delivered N.SF or SF on Response to Thermal Hypersensitivity in CIS-rats

Regarding the heat hypersensitivity test, CIS administration stimulated a significant increase in the response time by 392.76%, indicating a decrease in pain threshold in the tail-flick test, as compared to negative control rats. IN administration of either N.SF or SF to CIS-rats significantly decreased response time by 81.12% and 71.85%, respectively, as compared to CIS-rats, indicating that IN administration of N.SF or SF ameliorated pain threshold response. On the other side, N.SF-administrated rats showed an insignificant change to negative control rats. These findings indicated that the sensitivity to the thermal stimulus was increased in CIS-induced rats and that IN administration of either N.SF or SF ameliorated heat sensitivity in CIS-induced rats, as demonstrated by the tail-flick test. However, IN administration of N.SF was more potent in restoring thermal sensitivity than SF, as illustrated in Fig. (5).

Concerning the cold hypersensitivity test, both CIS-rats and N.SF-alone administrated rats demonstrated an insignificant change as compared to negative control rats. Similarly, IN administration of N.SF or SF to CIS-induced rats exhibited an insignificant change to CIS-rats, as demonstrated in Fig. (5).

### Effect of IN-Delivered N.SF or SF on Brain Acetylcholinesterase (AChE) Activities in CIS-Rats

The brain AChE activity in CIS-induced rats was significantly elevated by 50.65%, as compared to control rats. IN treatment of CIS-rats with either N.SF or SF enhanced a significant inhibition in AChE contents by 20.38% and 13.5%, respectively, as compared to CIS-induced rats. On the other side, N.SF-alone administrated rats showed an insignificant change to negative control rats. These findings showed that SF, either free or loaded, exhibited an anti-AChE potential, as illustrated in Fig. (6).

### Effect of IN-Delivered N.SF or SF on Oxidative Stress Status in CIS-Rats

**Fig. 7:**
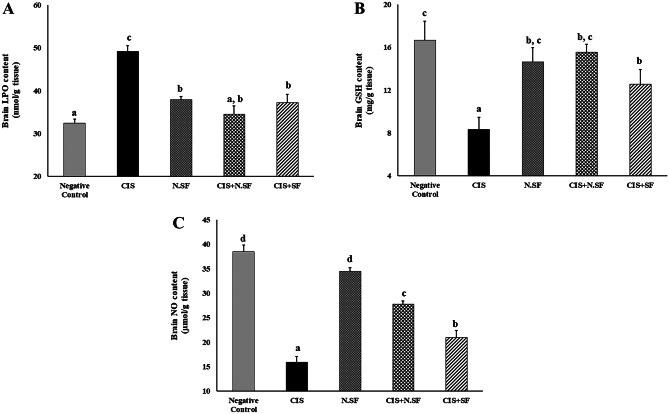
Effect of intranasal administration of sulforaphane (SF) loaded within iron oxide nanoparticles (N.SF) or free sulforaphane (SF) on brain contents of lipid peroxides (LPO), glutathione reduced (GSH), and nitric oxide (NO) in CIS-induced rats. Groups: negative control rats, cisplatin (CIS)-exposed rats, N.SF-alone exposed rats, CIS + N.SF-treated rats, and CIS + SF-treated rats. Results are presented as mean ± standard error of the mean (SEM). Mean with different letters (a–d) is significant at *p* ≤ 0.05

As compared to control rats, CIS-induced rats demonstrated a significant increase in lipid peroxides (LPO) by 51.85%. In contrast, brain contents of GSH and NO significantly declined by 50 and 58.75%, respectively. On the other hand, IN administration of either N.SF or SF significantly decreased LPO contents (29.68% and 24.25%, respectively) as well as significantly increased GSH and NO by 86.67% and 75.19%, respectively, for N.SF and 50.91% and 32.25%, respectively, for SF, as compared to CIS-rats. On the other side, N.SF-alone administrated rats demonstrated an insignificant change regarding NO, while exhibiting a mild increase of MDA and a moderate decline of GSH, as compared to negative control rats. These findings demonstrated the antioxidative potential of N.SF or SF against CIS-induced oxidative stress, as illustrated in Fig. 7(A–C).

### Effect of IN-Delivered N.SF or SF on Brain Distribution of Iron (Fe) and Platinum (Pt) in CIS-Brains

**Fig. 8:**
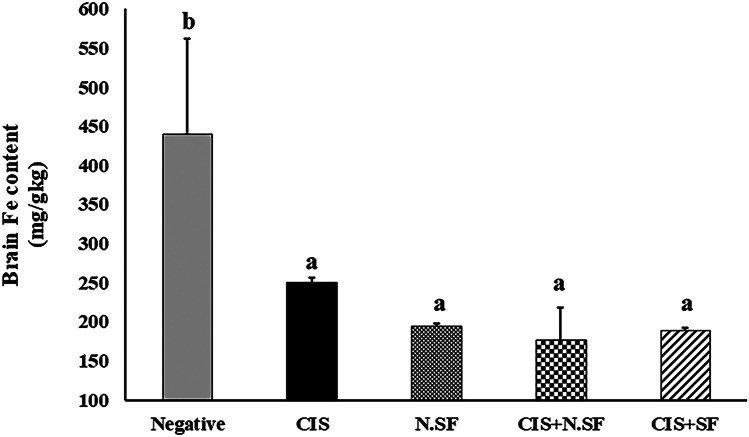
Iron (Fe) distribution in the brain of different experimental groups: negative control rats, cisplatin (CIS)-exposed rats, N.SF-alone exposed rats, CIS + N.SF-treated rats, and CIS + SF-treated rats. Results are presented as mean ± standard error of the mean (SEM). Mean with different letters (a–b) is significant at *p* ≤ 0.05

The brain contents of Fe or Pt were estimated using ICP-OES. Regarding brain Fe concentration, CIS-brains demonstrated a significant reduction of 43.18%, N.SF-brains exhibited a significant reduction by 55.68%, CIS + N.SF-treated brains displayed a significant reduction by 59.73%, and CIS + free SF-treated brains exhibited a significant reduction by 57%, as compared to the Fe content of negative control brains, as illustrated in Fig. 8. On the other hand, Pt concentration in the brain was undetectable by ICP-OES.

### Effect of IN-Delivered N.SF or SF on CIS-Induced Histopathological Alterations in the Brain Regions of:

#### Cerebral Cortex

**Fig. 9:**
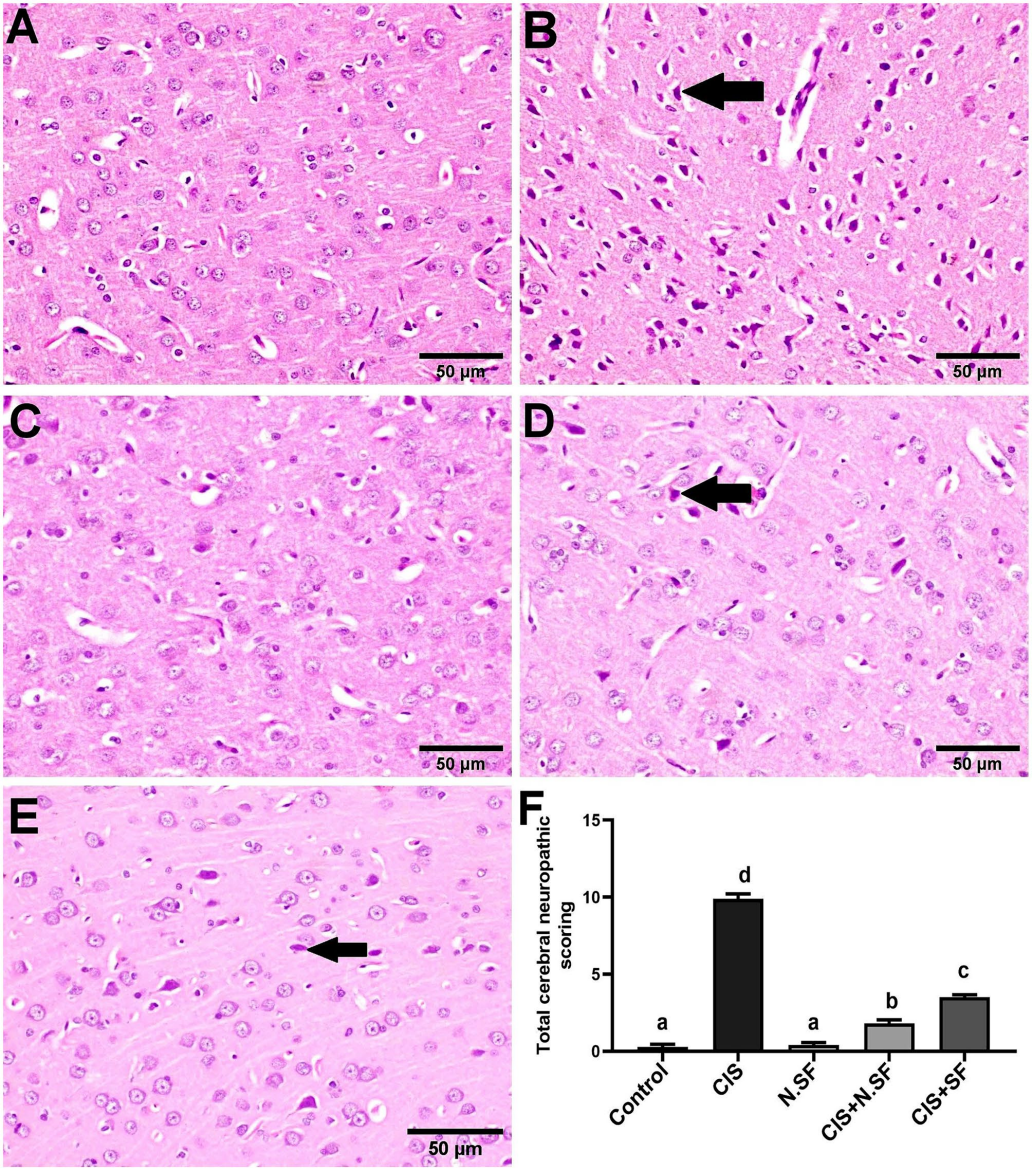
Representative photomicrographs of H&E-stained cerebral cortex of different experimental groups. **A** Negative control brains showing the normal histological architecture with intact neurons. **B** CIS-neurotoxicated brains showing prominent degeneration and necrosis of neurons (arrow). **C** N.SF alone-control brains showing no histopathological alterations. **D** CIS + N.SF-treated brains, showing necrosis of sporadic neurons (arrow). **E** CIS + SF-treated brains, showing necrosis of some neurons (arrow) (scale bar: 50 μm). **F** Total histological scoring of cerebral cortex damage. Results are presented as mean ± standard error of the mean (SEM). Mean with different letters (a–d) is significant at *p* ≤ 0.05

Microscopically, the cerebral cortex of negative control brains exhibited normal histological architecture with intact neurons (Fig. 9A). Contrariwise, prominent neuropathic lesions were investigated in the CIS-exposed cerebral cortex which was characterized by marked necrosis of neurons (Fig. 9B) associated with neuronophagia and gliosis. Meanwhile, the cerebral cortex of rats administered N.SF alone revealed normal intact neurons (Fig. 9C). Likewise, marked amelioration with regressed histopathological lesions was investigated in cerebral cortex of rats administered CIS + N.SF; treated brain tissue exhibited only necrosis of sporadic neurons (Fig. 9D). Meanwhile, cerebral cortex of rats administered CIS + SF revealed necrosis of some neurons and neuronophagia (Fig. 9E). Figure 9F illustrates the total histological lesion scores in the cerebral cortex of different experimental groups.

#### Striatum

**Fig. 10:**
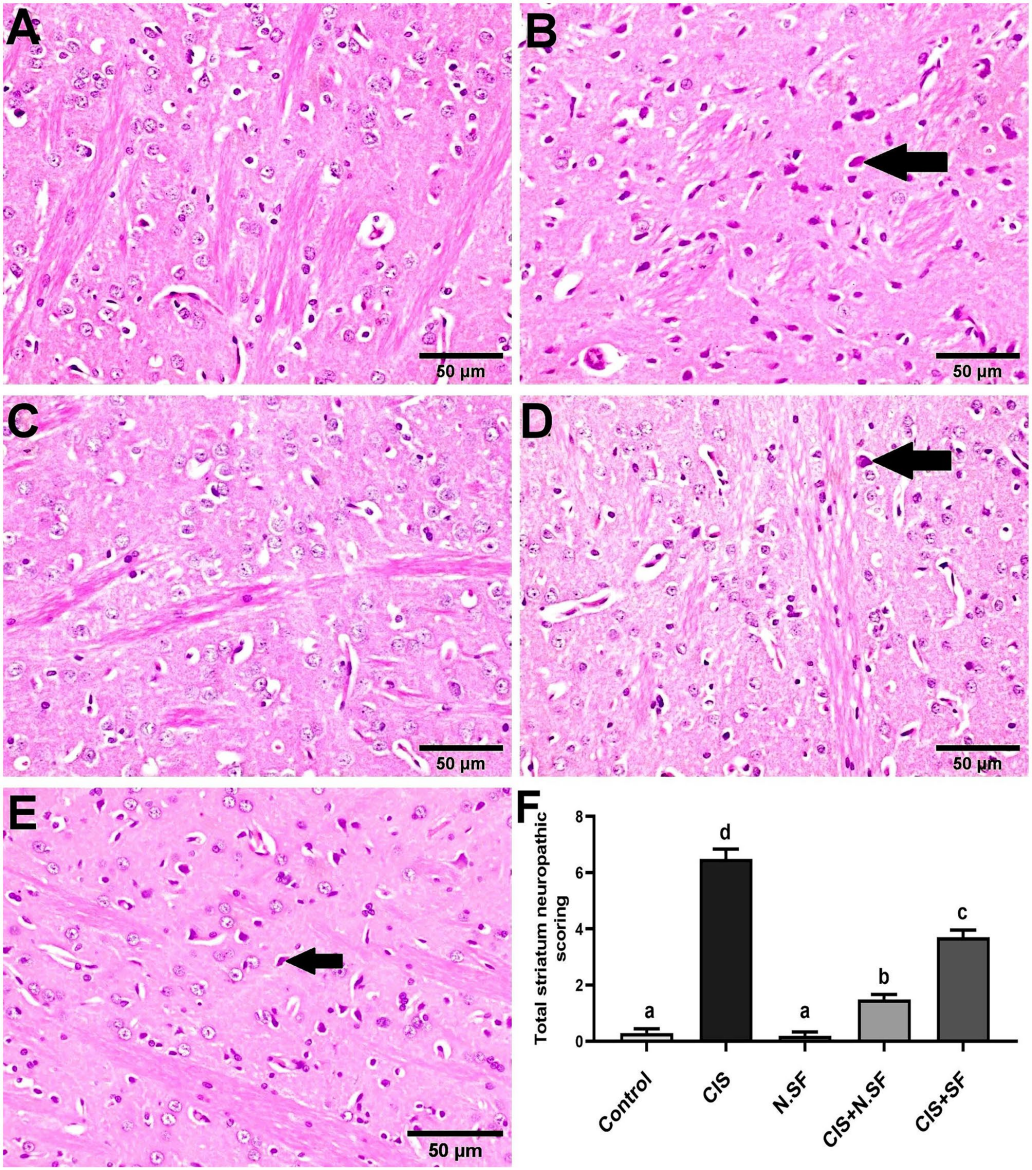
Representative photomicrographs of H&E-stained striata of different experimental groups. **A** Negative control brains showing the normal histological architecture with intact neurons. **B** CIS-induced brains showing necrosis of neurons (arrow). **C** N.SF-alone treated brains, showing no histopathological alterations. **D** CIS + N.SF-treated brains showing shrunken and necrosis of sporadic neurons (arrow). **E** CIS + SF-treated brains showing necrosis of some neurons (arrow) (scale bar: 50 μm). **F** Total histological scoring of striatum damage. Results are presented as mean ± standard error of the mean (SEM). Mean with different letters (a–d) is significant at *p* ≤ 0.05

Light microscopic examination of the striatum of negative control brains exhibited the normal histological architecture with intact neurons (Fig. 10A). On contrary, remarkable severe neuropathic damage was investigated in CIS-intoxicated rats, and prominent degeneration, necrosis of neurons (Fig. 10B), and proliferation of glia cells were recorded. Otherwise, striatal sections of N.SF alone-treated brains showed no histopathological alterations (Fig. 10C). Furthermore, a marked improved picture was seen in the striatum of brains treated with CIS + N.SF examined sections that exhibited shrunken and necrosis of sporadic neurons (Fig. 10D). On the other hand, the striatum of rats treated with CIS + SF manifested moderate improved picture characterized by necrosis of some neurons and mild proliferation of glia cells (Fig. 10E). Figure 10F illustrates the total histological lesion scores in the striata of different experimental groups.

#### Hippocampus

**Fig. 11:**
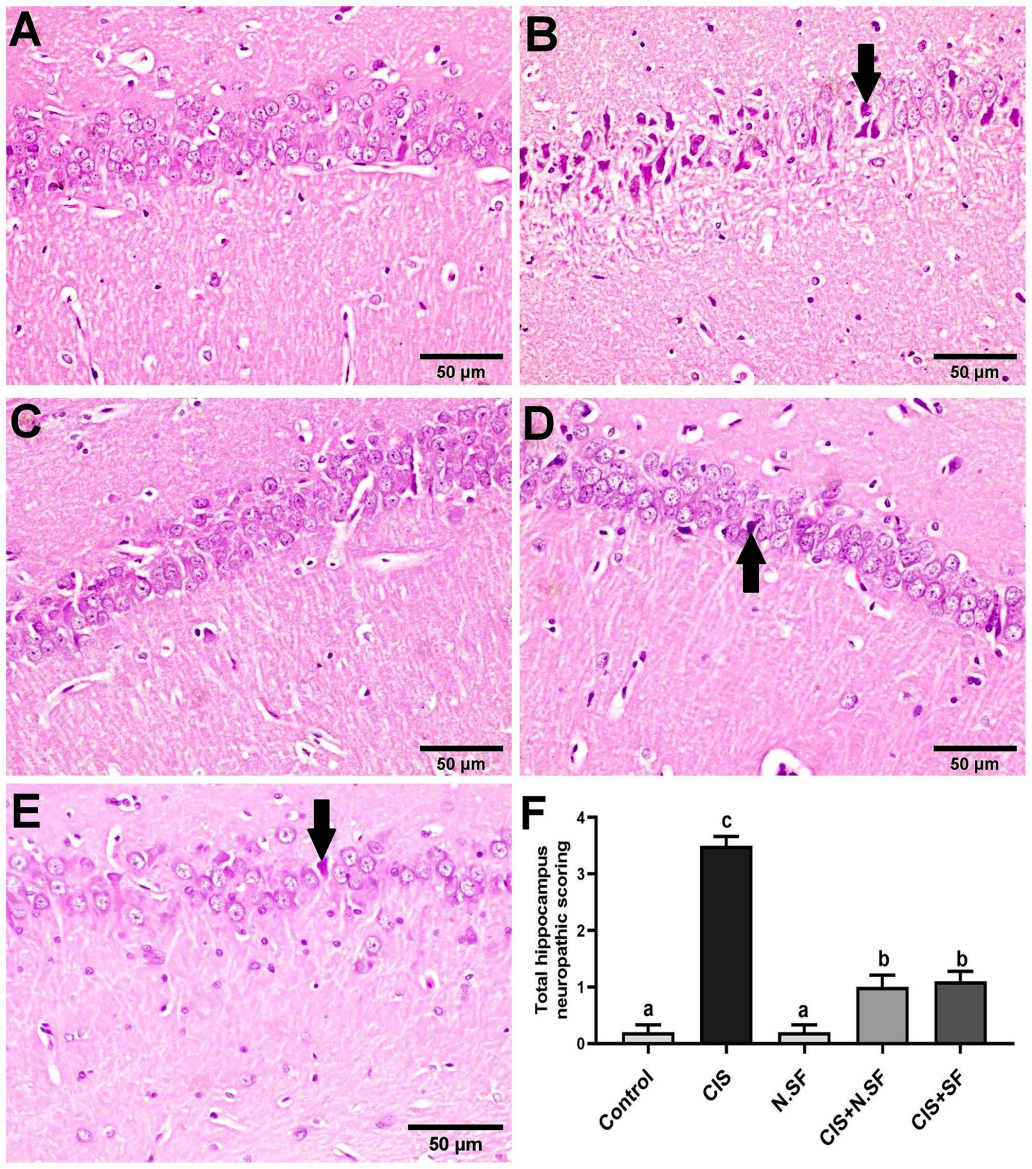
Representative photomicrographs of H&E-stained hippocampus of different experimental groups. **A** Negative control showing the normal histological architecture. **B** CIS-neurotoxicated brains, showing marked shrunken and pyknosis of pyramidal neurons (arrow). **C** N.SF-alone treated brains showing no histopathological changes. **D** and **E** CIS + N.SF- and CIS + SF-treated brains respectively showing sparse necrosis of pyramidal neurons (arrow) (scale bar: 50 μm). **F** Total histological scoring of hippocampus damage. Results are presented as mean ± standard error of the mean (SEM). Mean with different letters (a–c) is significant at *p* ≤ 0.05

A normal histological architecture of the hippocampus was observed in negative control brains (Fig. 11A). Otherwise, marked shrunken and pyknosis of pyramidal neurons (Fig. 11B) was demonstrated in CIS-neurotoxicated hippocampus. Meanwhile, administration of N.SF alone induced no histopathological changes in the hippocampus (Fig. 11C). On the other hand, CIS + N.SF and CIS + SF treatments resulted in a notable improvement. Hippocampus sections revealed more or less normal pyramidal neurons, whereas only sparse necrosis of pyramidal neurons was noticed (Fig. 11D and E). Figure 11F illustrates the total histological lesion scores in the hippocampi of different experimental groups.

#### Cerebellum

**Fig. 12:**
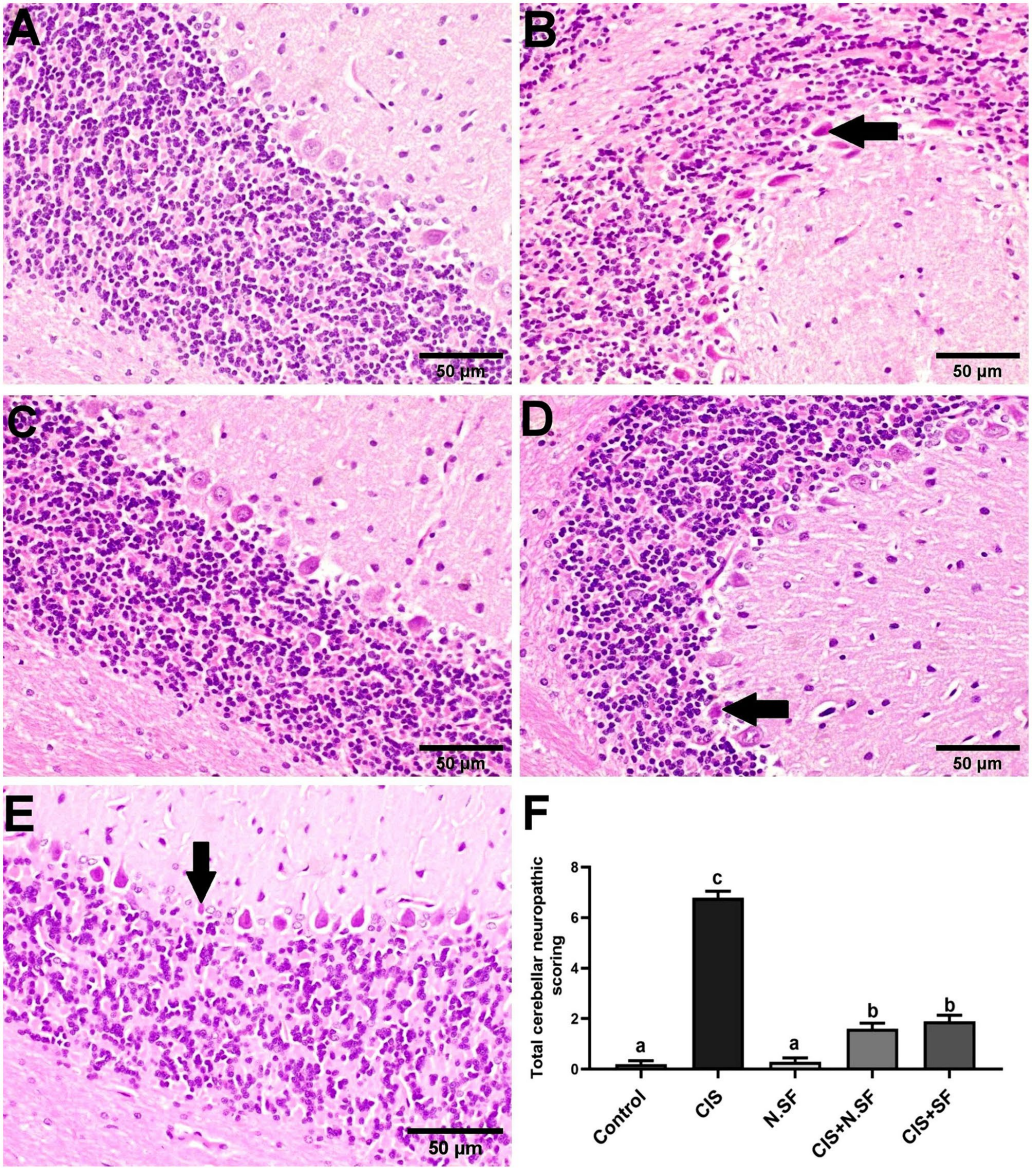
Representative photomicrographs of H&E-stained cerebellum of different experimental groups. **A** Negative control brains showing the normal histological architecture. **B** CIS-neurotoxicated brains showing pyknotic and shrunken Purkinje cells (arrow). **C** N.SF alone treated brains showing normal histological structure. **D** and **E** CIS + N.SF- and CIS + SF-treated brains, respectively, showing degeneration of sporadic Purkinje cells (arrow) (scale bar: 50 μm). **F** Total histological scoring of cerebellum damage. Results are presented as mean ± standard error of the mean (SEM). Mean with different letters (a–c) is significant at *p* ≤ 0.05

Microscopically, cerebellar tissue of the negative control brains showed normal histological architecture structure (Fig. 12A). Contrariwise, sections from CIS-induced brains exhibited pyknotic and shrunken Purkinje cells (Fig. 12B) and vacuolization of neuropil. However, N.SF alone-cerebellum revealed normal histological structure (Fig. 12C). The cerebellums of CIS + N.SF and CIS + SF treated groups showed a regression in the histopathological alterations, examined sections exhibited necrosis of sporadic Purkinje cells (Fig. 12D and E). Figure 12F illustrates the total histological lesion scores in the cerebellum of different experimental groups.

## Discussion

Recently, the non-invasive intranasal (IN) trafficking of therapeutic agents with nanocarriers has attracted a lot of attention (Don et al. 2021), as nanocarriers can enhance the efficacy of drug delivery, biodistribution, bioavailability, and absorption against enzymatic degradation and extracellular transportation by P-gp efflux proteins (Mistry et al. 2009; Pires et al. 2009). Accordingly, direct IN trafficking of nanocarriers is expected to reduce drug wastage, administration frequency, and undesirable adverse effects (Don et al. 2021). The choice of the nasal route for brain-targeted delivery of SF loaded within Fe_3_O_4_-NPs was based on the ability of the nasal route to improve the physicochemical stability of SF, and to enhance its bioavailability by avoiding oral route drawbacks like extensive first-pass metabolism and intestinal drug degradation (Salem et al. 2019). Based on our previous research (Mabrouk et al. 2021), we demonstrated that chloride Fe_3_O_4_-NPs exhibited a safe toxicological profile evidenced by physicochemical and biological investigations; therefore, we aimed to explore the nanotherapeutic potential of intranasally delivered SF loaded within Fe_3_O_4_-NPs (N.SF) against CIS-induced neurotoxicity through different biochemical, behavioral, and histological investigations. We used this formulation to deliver SF in the group that received only N.SF to affirm its safety profile.

Administration of a single i.p. dose of CIS disrupted neuromuscular coordination, resulted in muscle weakness, and decreased latency to fall in the hanging wire “grip” test. Our results run in accordance with Shabani et al. (2012a, b) and Wahdan et al. (2021). This might be ascribed to the neurotoxic potential of CIS to disrupt the neuronal function in the cerebellar cortex, especially Purkinje cells which are the essential part for movement control and motor functions (Pisu et al. 2004; Avella et al. 2006).

In addition, CIS administration provoked thermal hyperalgesia and stimulated sensitivity to pain, this runs in accordance with several studies (Ali et al. 2014; Sharawy et al. 2015; Ahmad et al. 2017; Saadati et al. 2021; Wahdan et al. 2021). CIS-induced thermal hyperalgesia might be attributed to the axonal deterioration in the sciatic nerve and the lower spine of the spinal cord, indicating the potential of CIS to affect small fibers that are closely involved in thermal hypersensitivity (Liu et al. 2022). However, our findings regarding cold hypersensitivity do not agree with Sharawy et al. (2015). This alteration in the behavioral outcome might be ascribed to variations in the used thermal stimulus and the used dose as well as the duration of CIS-induced neurotoxicity (Zhao et al. 2012; Al Moundhri et al. 2013).

Histopathologically, CIS-exposed cerebellar cortices displayed pyknotic and shrunken Purkinje cells; the histopathological examination of cerebellar cortices is strongly correlated with impaired neuromuscular functions. In addition, CIS altered the histological architecture of the striatum; the striatum is the input module to the “basal ganglia,” a neuronal circuit essential for voluntary movement control (Hikosaka et al. 2000); the basal ganglia interacted extensively with the cerebral cortex (Reiner et al. 1998). These histological alterations in the four different regions of the brain could be ascribed to CIS-induced oxidative stress, and explained the disrupted neuromuscular function and the enhanced thermal hyperalgesia in CIS-rats.

On the other side, treatment of CIS-induced rats with IN-delivered N.SF significantly ameliorated the neuromuscular strength and coordination as well as decreased sensitivity to the thermal stimulus, confirming the neuroprotective potential of loaded SF and the absence of neurotoxic impact of Fe_3_O_4_-NPs. In addition, the N.SF administration to CIS-rats enhanced a better behavioral outcome than that of SF; IN administration of either N.SF or SF to CIS-rats significantly increased the time elapsed for wire hanging by 405.78% and 126.2%, respectively, and significantly decreased response time in the tail-flick test by 81.12% and 71.85%, respectively, as compared to CIS-rats. The histopathological examination of different brain sections corroborated the behavioral findings that demonstrated the restored neuromuscular coordination and reflected the repair of both peripheral and central neural function.

Herein, IN-administrated N.SF exerted more neuroprotective potential than that of free SF; this was clearly observed histopathologically as the treatment with N.SF was more capable of mitigating both CIS-induced striatal and cortical injuries. While IN treatment with either SF or N.SF showed equal alleviative potential regarding CIS-induced hippocampal or cerebellar injury. It was suggested that the striatum, the cerebral cortex, and the cerebellum form network acting on multiple motor and non-motor activities (Milardi et al. 2019). In addition, the striatum receives a massive input from the cerebral cortex (Wichmann and DeLong 2008), and is thought to provide instructions to the cortex (Graybiel 2005). In rodents, cortico-spinal pyramidal neurons directly synapse on the striatum, along with the initial signal to the striatum; the cerebral cortex inhibits surrounding or competing motor patterns (Milardi et al. 2019). Therefore, the superior therapeutic potential of N.SF rather than that with SF, as manifested by behavioral and histopathological findings, might be ascribed to the neuroprotective activities in the four different regions of the brain mainly the regions of striatum and cerebral cortex.

AChE is one of the crucial enzymes responsible for neurotransmission (Bugata et al. 2019). The cholinergic function seems to be vulnerable to CIS-provoked neurotoxicity; CIS-rats exhibited elevated brain AChE activities that contributed to hydrolysis of acetylcholine and its scarcity at the synaptic connections (Fouad 2020). Activation of AChE could lead to cholinergic hyperactivity and neurocognitive impairment, and might be partly associated with oxidative stress (Borai et al. 2017; Ibrahim Fouad and Ahmed 2021). In addition, CIS administration elevated serum, brain, and renal levels of “indoxyl-sulfate” which is a uremic toxin that is capable of stimulating oxidative stress and neurotoxicity (Adesso et al. 2017).

Actually, the different brain regions such as the striatum exhibited a greater vulnerability to oxidative stress, which was indicated by significantly elevated lipid peroxidation (51.85%) as well as significantly declined brain contents of GSH and NO (50% and 58.75%, respectively) in CIS-exposed brains. CIS-induced ROS generation disrupts mitochondrial function and integrity, disrupts BBB, and inhibits the proliferation of neuronal stem cells (NSCs) (Kütük et al. 2019; Liu et al. 2022). Additionally, CIS impairs antioxidant defense efficiency and provokes an imbalance in the pro-oxidant/antioxidant system (Yadav 2019). CIS-induced oxidative stress is strongly correlated with neuroinflammation and results in neural shrinkage and axonal demyelination leading to slow impulse conduction (Manohar et al. 2014; Wahdan et al. 2021), explaining CIS-associated behavioral alterations and motor impairments.

The GSH depletion in CIS-induced brains could be attributed to the uptake of the formed “Pt-sulphydryl group complexes” by neuronal cells and its rapid transformation to reactive metabolites. The subsequent GSH depletion contributes to enhancing the neurotoxic potential of CIS and mediates lipid peroxidation and impairment of the antioxidant defense system (Majd et al. 2021). Similarly, the NO depletion in CIS-induced brains could be explained by the potential of CIS to inhibit neuronal nitric oxide synthase (nNOS) “NO-producing enzyme” that is Ca^2+^/calmodulin-dependent; CIS interacts with Ca^2+^-binding sites of gastric calmodulin and results in indirect suppression of nNOS activity (Jarve and Aggarwal 1997). Therefore, it could be deduced that a similar mechanism might take place in CIS-induced brains resulting in NO depletion, as explained by Gulec et al. (2013). Depletion of brain NO could provoke endothelial dysfunction, decrease cerebral blood flow (CBF), and boost early brain injury (Garry et al. 2015; Kütük et al. 2019; Fouad 2020). Therefore, further research work is required to investigate the underlying mechanisms of NO role in CIS-induced neurotoxicity.

It was observed that N.SF-brains displayed a mild increase of MDA (17.28%) along with a moderate reduction of GSH (12%), as compared to negative control brains. This moderate-induced oxidative stress might be ascribed to a normal and physiological enhancement of “redox defenses” against Fe_3_O_4_-NPs, used as nanotherapeutics. Therefore, the brain deposition of Fe_3_O_4_-NPs should be carefully monitored to avoid the development of nanotoxicity. Notably, no pathological alterations were found in the N.SF-exposed different brain regions, as compared to control brains. Hence, it is speculated that the slight elevation in redox biomarkers does not influence cellular integrity or tissue structure in the brain. This runs in agreement with our previous study (Mabrouk et al. 2021).

Conversely, IN trafficking of either N.SF or SF demonstrated antioxidative and anti-AChE activities; by decreasing lipid peroxidation and elevating GSH and NO, and ameliorating cholinergic function through inhibiting AChE activities, as compared to CIS-brains. This neuroprotective potential of either N.SF or SF impact might be ascribed to the potential of SF “Nrf2 activator” to rapidly cross the BBB, resulting in an increment in the genetic expression of Nrf2 in the striatum and cortex 1–4 h after administration (Jazwa et al. 2011). Our findings demonstrated that N.SF presented better antioxidative and anti-AChE activities than those of SF; this superior neuroprotective potential of N.SF was reflected in the improvement of neuromuscular coordination and the reduced thermal hyperalgesia.

The superior neuroprotective potential of N.SF is largely attributed to the increase in the size of Fe_3_O_4_-NPs after drug loading, as demonstrated by TEM results, where the increase of drug (SF) loading accompanied by a larger volume of the pores results in increasing average sizes of the nanoparticles (Huang et al. 2018). However, the recorded particle size distribution demonstrated very close values to those reported for Fe_3_O_4_-NPs alone without the drug (Mabrouk et al. 2021), which indicates a great encapsulation of SF within the pores of Fe_3_O_4_-NPs. The results of zeta potential illustrate a negative zeta potential of the prepared drug-loaded Fe_3_O_4_-NPs. Moreover, the original particle charge of Fe_3_O_4_-NPs was − 14 mV which tends to lower negativity in the presence of SF, which reflects the influence of Fe_3_O_4_-NPs as “SF carriers.” The results of zeta potential illustrate a negative zeta potential of the prepared SF-loaded Fe_3_O_4_-NPs. This negative charge induces cell internalization. In addition, the short duration of IN administration of N.SF (10 days) was aimed to reduce the in vivo exposure to Fe_3_O_4_-NPs to avoid stimulation of oxidative stress and neuroinflammation, as long-term deposition of NPs in the brain might contribute to cellular interaction and ROS generation which could trigger neurotoxicity (Sun et al. 2011). Wu et al. (2013) found that more than half of the Fe_3_O_4_-NPs remained in the striatum and hippocampus 14 days post-instillation after intranasal instillation and enhanced oxidative damage to the striatum. Therefore, the IN of N.SF lasted for less than 14 days to avoid neurotoxicity and deposition of Fe_3_O_4_-NPs. Herein, N.SF + CIS brains showed better histological improvement in the four examined brain regions, including the striatum and the cerebral cortex, than that of SF + CIS brains.

Furthermore, iron (Fe) and platinum (Pt) content in the brain tissues was assessed. The non-detectable Pt concentration in the brain might be ascribed to the inability of CIS to cross BBB (Breglio et al. 2017). Despite its inability to cross BBB, CIS is capable of disturbing Fe distribution in the brain and thus affecting the Fe homeostasis in the brain; CIS-brains showed a significant reduction in Fe content (43.18%), as compared to negative brains. Additionally, oxidative stress and neuroinflammation might dysregulate Fe homeostasis and collaborate to alterations observed in the pathophysiology of Parkinson’s disease (PD) (Medeiros et al. 2016). “Fe” plays an essential role in the synthesis of neurotransmitters and acts as a cofactor of tyrosine hydroxylase; thus, the decreased Fe content in CIS-brains is linked to the malfunctioning of neuronal enzymes by contributing to a reduced Fe storage in neurons (Pichler et al. 2013). In addition, Fe deficiency might disturb mitochondrial efficiency and stimulate oxidative stress in the brain (Jeong et al. 2011; Wan et al. 2012). For instance, the declined NO might enhance Fe deprivation by reducing Fe export from the substantia nigra (SN) to the periphery (Younes-Mhenni et al. 2007); this explains the cause of reduced Fe and NO contents in CIS-brains. However, further studies should be conducted to investigate this point.

On the other side, N.SF-brains, CIS + N.SF-treated brains, and CIS + SF-treated brains displayed a significant reduction by 55.68, 59.73, and 57%, respectively, as compared to Fe content of negative brains; this might be explained by the effective neurotherapeutic potential of SF-loaded Fe_3_O_4_-NPs that were not deposited in the brain and accordingly did not enhance oxidative stress; Fe_3_O_4_-NPs are biocompatible and biodegradable, and can be incorporated into the body’s Fe cycle upon degradation (Liu et al. 2016). Therefore, we could assume that loaded SF neutralized neuroinflammation and oxidative stress and improved BBB integrity, which might be involved in less Fe deposition in the brain. Finally, we could deduce that loading SF within Fe_3_O_4_-NPs potentiates the neuroprotective and antioxidant activities of SF.

## Conclusion

IN administration of either N.SF or SF was able to negate CIS-induced oxidative stress and neurotoxicity; however, N.SF showed superior neuroprotective potential against CIS-induced peripheral and central neurotoxicity than that of SF. This was supported by the behavioral, biochemical, and histopathological findings. Therefore, IN administration of N.SF could represent an alternative, simple, and highly compliant approach to managing CIS-associated neurotoxicity. Further studies are required to elucidate the mechanisms underlying this effect and to identify potential biomarkers related to peripheral and/or central neurotoxicity. Additionally, these encouraging results demonstrated the potential use of iron-oxide NPs as neurotherapeutic agents and confirmed the possibility of developing a novel promising and non-invasive intranasal delivery system for the treatment of CIS-induced neurotoxicity. Once translated clinically, the IN administration of this novel nanotheranostic formulation will be a valuable tool to potentiate the neuroprotective impact of SF in CIS-receiving patients and improve their quality of life.

## Data Availability

All data generated or analyzed during this study are included in this published article.

## Abbreviations

CIS: Cisplatin
SF: Sulforaphane
AChE: Acetylcholinesterase
LPO: Lipid peroxides
MDA: Malondialdehyde
GSH: Glutathione reduced
NO: Nitric oxide
Fe: Iron
Pt: Platinum
BBB: Blood-brain barrier
PN: Peripheral neuropathy
Fe_3_O_4_-NPs: Iron oxide nanoparticles
IO: Iron oxide
N.SF: Sulforaphane-loaded Fe_3_O_4_-NPs
TEM: Transmission electron microscopy
IN: Intranasal
ICP-OES: Inductively coupled plasma optical emission spectrometer
NSCs: Neuronal stem cells
Nrf2: Nuclear factor erythroid 2-related factor 2
ROS: Reactive oxygen species
